# Application of Isokinetic Dynamometry Data in Predicting Gait Deviation Index Using Machine Learning in Stroke Patients: A Cross-Sectional Study

**DOI:** 10.3390/s24227258

**Published:** 2024-11-13

**Authors:** Xiaolei Lu, Chenye Qiao, Hujun Wang, Yingqi Li, Jingxuan Wang, Congxiao Wang, Yingpeng Wang, Shuyan Qie

**Affiliations:** 1 Department of Rehabilitation, Beijing Rehabilitation Hospital, Capital Medical University, Beijing 100144, China; 18853639611@163.com (X.L.); hujunwang@ccmu.edu.cn (H.W.); lyq9874@163.com (Y.L.); 2 Beijing Rehabilitation Medicine, Beijing Rehabilitation Hospital, Capital Medical University, Beijing 100144, China; qcy0424@mail.ccmu.edu.cn (C.Q.); wangjingxuanella@126.com (J.W.)

**Keywords:** sensors, isokinetic dynamometry, gait deviation index, machine learning, stroke

## Abstract

Background: Three-dimensional gait analysis, supported by advanced sensor systems, is a crucial component in the rehabilitation assessment of post-stroke hemiplegic patients. However, the sensor data generated from such analyses are often complex and challenging to interpret in clinical practice, requiring significant time and complicated procedures. The Gait Deviation Index (GDI) serves as a simplified metric for quantifying the severity of pathological gait. Although isokinetic dynamometry, utilizing sophisticated sensors, is widely employed in muscle function assessment and rehabilitation, its application in gait analysis remains underexplored. Objective: This study aims to investigate the use of sensor-acquired isokinetic muscle strength data, combined with machine learning techniques, to predict the GDI in hemiplegic patients. This study utilizes data captured from sensors embedded in the Biodex dynamometry system and the Vicon 3D motion capture system, highlighting the integration of sensor technology in clinical gait analysis. Methods: This study was a cross-sectional, observational study that included a cohort of 150 post-stroke hemiplegic patients. The sensor data included measurements such as peak torque, peak torque/body weight, maximum work of repeated actions, coefficient of variation, average power, total work, acceleration time, deceleration time, range of motion, and average peak torque for both flexor and extensor muscles on the affected side at three angular velocities (60°/s, 90°/s, and 120°/s) using the Biodex System 4 Pro. The GDI was calculated using data from a Vicon 3D motion capture system. This study employed four machine learning models—Lasso Regression, Random Forest (RF), Support Vector regression (SVR), and BP Neural Network—to model and validate the sensor data. Model performance was evaluated using mean squared error (MSE), the coefficient of determination (R^2^), and mean absolute error (MAE). SHapley Additive exPlanations (SHAP) analysis was used to enhance model interpretability. Results: The RF model outperformed others in predicting GDI, with an MSE of 16.18, an R^2^ of 0.89, and an MAE of 2.99. In contrast, the Lasso Regression model yielded an MSE of 22.29, an R^2^ of 0.85, and an MAE of 3.71. The SVR model had an MSE of 31.58, an R^2^ of 0.82, and an MAE of 7.68, while the BP Neural Network model exhibited the poorest performance with an MSE of 50.38, an R^2^ of 0.79, and an MAE of 9.59. SHAP analysis identified the maximum work of repeated actions of the extensor muscles at 60°/s and 120°/s as the most critical sensor-derived features for predicting GDI, underscoring the importance of muscle strength metrics at varying speeds in rehabilitation assessments. Conclusions: This study highlights the potential of integrating advanced sensor technology with machine learning techniques in the analysis of complex clinical data. The developed GDI prediction model, based on sensor-acquired isokinetic dynamometry data, offers a novel, streamlined, and effective tool for assessing rehabilitation progress in post-stroke hemiplegic patients, with promising implications for broader clinical application.

## 1. Introduction

Stroke remains a leading cause of disability and mortality worldwide, with a rising incidence and recurrence rates [1]. It primarily affects the central nervous system, often resulting in long-term motor dysfunction, particularly in the lower limbs, which frequently manifests as gait abnormalities [2,3]. These gait impairments not only impede patients’ daily activities but are also strongly correlated with increased risks of falls, recurrent strokes, and other complications [4,5,6]. Consequently, these challenges impose significant physical and psychological burdens on patients and present substantial economic strains on families and society. Gait analysis is a critical component of rehabilitation assessment in stroke patients, providing precise kinematic data to inform personalized rehabilitation strategies [7,8]. Accurate gait assessment facilitates not only the monitoring of recovery progress but also the prediction of future fall risks and potential secondary injuries, thereby guiding clinical interventions more effectively [9]. Research indicates that the precision of gait analysis is directly linked to rehabilitation outcomes, underscoring its indispensable role in stroke rehabilitation [10].

Traditional gait assessment methods often rely on clinical visual observations or basic physical measurements, such as gait speed and stride length [11]. While these methods are straightforward and practical, they are prone to subjective biases and fail to capture the complexity of gait patterns [12]. Despite the adoption of modern gait analysis tools, such as three-dimensional motion capture systems and portable gait analysis devices, these systems are often costly, complex to operate, and require specific equipment and environments, limiting their broader clinical application [13,14]. Isokinetic dynamometry, a widely used tool in sports medicine and rehabilitation, accurately measures muscle strength and function, providing vital data on muscle performance during dynamic tests [15]. However, despite the extensive data generated by isokinetic assessments, their potential for gait prediction remains underutilized.

With advances in computational capabilities and the development of big data technologies, machine learning has achieved significant progress in the medical field in recent years. These algorithms are extensively applied in medical imaging analysis, disease prediction, and personalized treatment planning due to their strengths in managing large-scale and complex datasets [16,17,18]. In stroke rehabilitation, machine learning can extract valuable features from multimodal data, enhancing diagnostic and therapeutic accuracy [16]. Integrating isokinetic muscle strength data with machine learning algorithms for gait prediction can effectively address the limitations of traditional methods. Preliminary studies have demonstrated the significant potential of machine learning models in processing biomechanical data, enabling the extraction of key features that influence gait [19]. This approach not only improves prediction accuracy but also facilitates the early detection of gait deviations, allowing for more targeted rehabilitation interventions for stroke patients. Previous studies have made significant progress in gait analysis and prediction using machine learning techniques, particularly deep learning approaches that have shown promise in classifying and predicting gait in stroke patients [20,21,22]. However, much of this research has focused on analyzing imaging data or simple gait parameters, with relatively few studies incorporating muscle strength data into gait prediction. Meanwhile, existing models often struggle with generalization when dealing with complex gait features, and the lack of diversity in data samples limits their applicability [23]. In addition, the accuracy of current gait prediction models in identifying gait deviations needs improvement, particularly when dealing with complex movement patterns [24].

This study primarily aims to analyze isokinetic muscle strength data using machine learning algorithms to predict the Gait Deviation Index (GDI) in stroke patients. This work seeks to fill gaps in existing research and provide a novel tool for gait assessment, further optimizing rehabilitation strategies for stroke patients. By developing more accurate gait prediction models, this study aims to make progress in improving the precision and clinical feasibility of gait analysis, thereby enhancing stroke rehabilitation programs and providing valuable references for future research in related fields.

## 2. Materials and Methods

### 2.1. Study Design and Participant Selection

The primary hypothesis of this study is that sensor-acquired isokinetic muscle strength data can significantly predict the GDI in stroke patients, leading to improved rehabilitation strategies. This study was designed as a cross-sectional, observational study involving a cohort of 150 post-stroke hemiplegic patients (91 males and 59 females) who were recruited from the Department of Rehabilitation, Beijing Rehabilitation Hospital, Capital Medical University. The recruitment process was overseen by the principal investigator, Congxiao Wang, MD, PhD, and a dedicated research team, ensuring that all ethical standards and procedures were adhered to throughout this study. The inclusion criteria were as follows: (1) age between 18 and 75 years; (2) a confirmed diagnosis of post-stroke hemiplegia with a disease duration of more than 3 months; (3) the ability to walk independently for at least 10 m; and (4) normal cognitive function with a Mini-Mental State Examination (MMSE) score of ≥24 points [25]. Exclusion criteria were as follows: (1) the presence of other neurological diseases affecting gait; (2) severe cardiovascular or other systemic diseases; (3) a history of lower limb surgery within the past 6 months; and (4) severe bone or joint diseases. Withdrawal criteria included the following: (1) occurrence of serious adverse events; (2) failure to complete the assessment as per the established protocol; and (3) voluntary withdrawal.

To assess the representativeness of our sample, we conducted a statistical comparison between the study group and reference values from the literature [26]. The characteristics compared included age distribution, gender ratio, residence distribution, hypertension rate, and diabetes rate. A chi-square test was applied to each characteristic to examine any significant differences.

This study was approved by the Ethics Committee of Beijing Rehabilitation Hospital, Capital Medical University (Ethics Approval Number: 2022bkky-029). All participants provided written informed consent before participating in the study, which detailed the research process, potential risks, and participants’ rights. The testing procedures were thoroughly explained to the participants, who were allowed to withdraw from the study at any time without consequences.

### 2.2. Experimental Design and Data Collection

**Figure 1:**
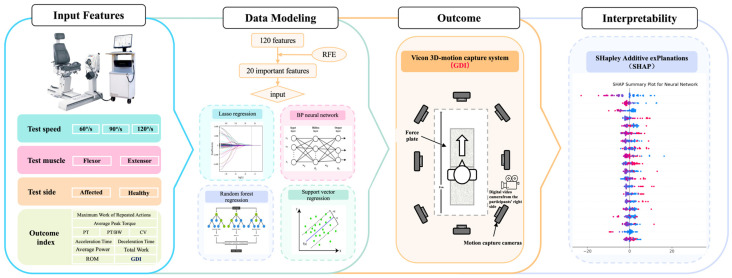
Overview of the proposed pipeline. A set of 20 features, selected through RFE—including test speed (3 features), muscle tests (2 features), test side (2 features), and outcome metrics (10 features)—were used as inputs to four distinct data modeling techniques: Lasso Regression, BP Neural Network model, RF Regression, and SVR. The objective was to predict the GDI score obtained via the Vicon motion capture system. Hyperparameter optimization was performed using 10-fold cross-validation. Finally, to provide clear interpretability of the results, the SHAP technique was employed and compared.

**Figure 2:**
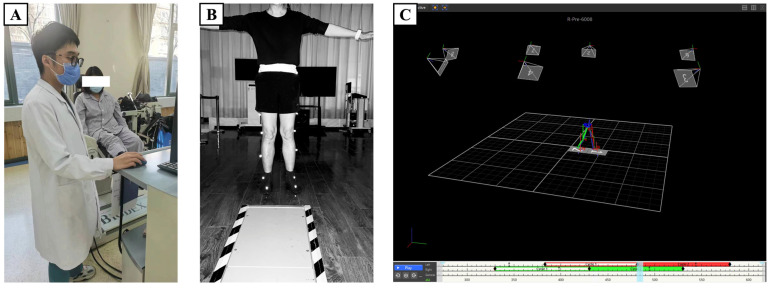
(**A**) Isokinetic dynamometry procedure; (**B**) Vicon 3D gait analysis static calibration; (**C**) software calculation procedure.

The general workflow of our study is displayed in Figure 1. All participants underwent two standard clinical tests to assess their knee joint isokinetic muscle strength and GDI. To ensure consistency in the testing process and data accuracy, all tests were conducted by professional rehabilitation therapists. Initially, muscle strength of the bilateral knee joints was evaluated using the Biodex System 4 Pro device (Shirley, NY, USA), as shown in Figure 2A. During testing, participants were seated on the Biodex device chair, with their back and thighs secured to minimize interference from other body parts. Each speed test was performed five times, with the average value taken as the final result. Participants were required to warm up adequately before each test to reduce the risk of injury and enhance test accuracy. Peak torque (PT), peak torque/body weight (PT/BW), the maximum work of repeated actions, coefficient of variation (CV), average power, total work, acceleration time, deceleration time, range of motion (ROM), and average peak torque were measured for both flexor and extensor muscles at speeds of 60°/s, 90°/s, and 120°/s. Compensation from other body parts, such as the trunk and upper limbs, was not allowed during the test. Subsequently, GDI data were collected and analyzed using the Vicon three-dimensional motion capture system (MX T40-S cameras, Oxford Metrics Limited, Oxford, UK) in the gait laboratory. The Vicon system was calibrated and validated before testing to ensure data capture accuracy, as shown in Figure 2B. Participants wore tight, non-black, non-reflective clothing to minimize capture errors. This study employed the Plugs-in-gait lower limb model, with 16 reflective markers placed on key anatomical landmarks to establish a lower limb model during walking. Before the test, participants walked back and forth naturally on an 8 m walkway to acclimate to the experimental environment. During the test, static testing was first conducted to determine the static model, followed by data collection as participants walked back and forth at a comfortable speed on the walkway. GDI for the affected side was automatically calculated after data processing with Vicon Nexus 2.12 software, including filling, smoothing, and filtering, as depicted in Figure 2C.

### 2.3. Data Preprocessing and Analysis

After data collection, data preprocessing was performed using Python 3.11 to ensure data consistency and accuracy. The steps included the following: (1) Normalization: *Z*-score normalization was applied to standardize the data by subtracting the mean and dividing by the standard deviation, making each feature comparable across different units and scales. (2) Outlier Handling: Outliers were identified using the Interquartile Range (IQR) method, and adjustments were made to minimize their impact on model performance. (3) Missing Data Imputation: Multiple imputation was applied to manage missing values, preserving data integrity and reducing potential biases. (4) Recursive Feature Elimination (RFE): To refine the feature set, we used RFE with an L1-regularized linear regression model to select the 20 most predictive features. This method iteratively removed the least important features, aiming to reduce dimensionality and improve model efficiency. The selected features were renamed to Feature1, Feature2, …, Feature20, and a feature mapping table was created. Subsequently, various machine learning regression methods, including Lasso Regression, the backpropagation (BP) Neural Network model, Random Forest Regression (RF), and Support Vector Regression (SVR), were used to train models for predicting the GDI in stroke patients. The Lasso Regression model, which employs L1 regularization, is effective for feature selection and handling multicollinearity. Given the high dimensionality of our data, which involves multiple muscle function indicators and potential correlations between features, Lasso Regression can effectively reduce redundant features, retaining the most representative ones to improve model interpretability. Moreover, the sparsity of coefficients in the Lasso model helps reduce the impact of noise, enhancing model robustness and predictive performance [27]. The BP Neural Network model possess strong nonlinear modeling capabilities, achieving high performance in learning complex data patterns. Given the highly nonlinear characteristics of our data, the BP Neural Network model can capture the potential complex relationships between muscle strength data and GDI. We configured the BP Neural Network model with four hidden layers, each containing 12 neurons, to enhance its learning ability. The Rectified Linear Unit (ReLU) was used as the activation function to ensure effective model training and prevent vanishing gradient issues. Additionally, the Adam optimizer, with a low learning rate and appropriate batch size, was selected to improve model stability and convergence. Although BP Neural Network models are prone to overfitting on small datasets, careful hyperparameter tuning allowed our BP Neural Network model to achieve stable results during training [28]. The RF model is an ensemble method based on decision trees, enhancing the model’s generalization capability and significantly reducing the risk of overfitting. The RF model is well suited for high-dimensional, complex data and performs well when dealing with nonlinear relationships and feature interdependencies. Given the multidimensional muscle strength parameters and movement features in our dataset, RF’s automatic feature selection and ability to capture nonlinear relationships provide strong support for accurate GDI prediction. Additionally, the RF model is highly robust in managing noisy data, making it widely applicable in medical and biomechanical data analysis [29]. The SVR model is well suited for small-sample, high-dimensional data and uses a kernel function (the Radial Basis Function (RBF) in this study) to capture complex nonlinear relationships. Given the relatively small sample size and high feature dimensionality in this study, SVR demonstrates strong generalization ability in this context. Furthermore, SVR’s ability to balance model fitting and tolerance through regularization parameter C and smoothness parameter ϵ makes it suitable for the precise modeling of feature patterns in small datasets. The SVR model is an effective model choice for handling complex, multidimensional biomechanical data without requiring large sample sizes, providing a valuable option for GDI prediction in this study [30].

In the RFE method, based on a linear regression model with L1 regularization, 20 important features were selected, with 10 iterations and a step size of 2 for each iteration. For the Lasso Regression model, the regularization strength parameter α was set to 0.01, the maximum number of iterations was 2000, and the random seed was fixed at 21 to ensure reproducibility of the results. The BP Neural Network model used four hidden layers, each with 12 neurons, the ReLU activation function, Adam optimizer, a learning rate of 0.0005, a batch size of 64, and 300 training epochs. The RF model consisted of 150 decision trees with a maximum depth of 20 and a minimum sample split of 5, with the random seed also set at 21. For the SVR model, the Radial Basis Function kernel was used, with a regularization parameter C set at 0.5, ε set at 0.05, and a maximum of 2000 iterations. The hyperparameters for the models are shown in Table 1.

This study employed data splitting and cross-validation techniques. The dataset was split into a training set (70%) and a validation set (30%). For each model, 10-fold cross-validation was used on the training set to enhance robustness and prevent overfitting: (1) 10-fold cross-validation. The training data were divided into 10 equal subsets. Each subset served as a validation set once, while the remaining nine subsets were used for training. The process was repeated 10 times, and the results were averaged to provide stable performance metrics. (2) Hyperparameter Tuning. Hyperparameter optimization was conducted for each model to ensure optimal performance. Grid search was used to find the best parameters for each algorithm: For Lasso Regression, we optimized the regularization strength parameter α. For the Random Forest model, we tuned the number of trees, maximum depth, and minimum samples required for a split. For SVR, we adjusted the kernel type and the regularization parameter C and epsilon (ϵ). The BP Neural Network model’s architecture was fine-tuned by adjusting the number of hidden layers, neurons per layer, learning rate, and batch size. (3) Evaluation Metrics. The models’ performance was assessed using mean squared error (MSE), mean absolute error (MAE), and the coefficient of determination (R^2^). These metrics were averaged over the 10 folds, providing reliable indicators of each model’s accuracy, consistency, and generalizability.

### 2.4. Interpretability Techniques

To enhance the interpretability of the models, particularly when dealing with black-box models, this study utilized SHAP (SHapley Additive exPlanations) techniques. SHAP is a method for explaining machine learning model predictions, based on the Shapley value from cooperative game theory [31]. It decomposes the influence of each feature into contributions to the prediction, thereby determining the importance of each feature. This approach is valuable for identifying features that have a positive or negative impact on model outputs, which is crucial for further improving diagnostic models.

## 3. Results

The representativeness analysis demonstrated that the age, gender, residence, hypertension, and diabetes distributions in our study group were consistent with those reported in the literature. The age distribution was as follows: 40–49 years (24.7%), 50–59 years (19.3%), 60–69 years (17.3%), 70–79 years (5.3%), and 80 years and above (2.0%), with a chi-square *p*-value of 0.82, indicating no significant difference from the literature values. Gender distribution was also comparable, with 56.7% males and 43.3% females (*p* = 0.16). Similarly, residence distribution (urban: 58.7%, rural: 41.3%; *p* = 0.75), hypertension rate (36.7%; *p* = 0.78), and diabetes rate (12.0%; *p* = 0.38) showed no significant deviations from the reference values. These results affirm that our study sample is representative of the general population of post-stroke hemiplegic patients, supporting the generalizability of our findings. Details are shown in Table 2.

In this study, data on knee joint isokinetic muscle strength and GDI were collected and analyzed from a total of 150 post-stroke hemiplegic patients. Table 3 provides a summary of the key parameters measured for the flexor and extensor muscles on both sides at different speeds (60°/s, 90°/s, 120°/s), including the median values and IQR.

**Figure 3:**
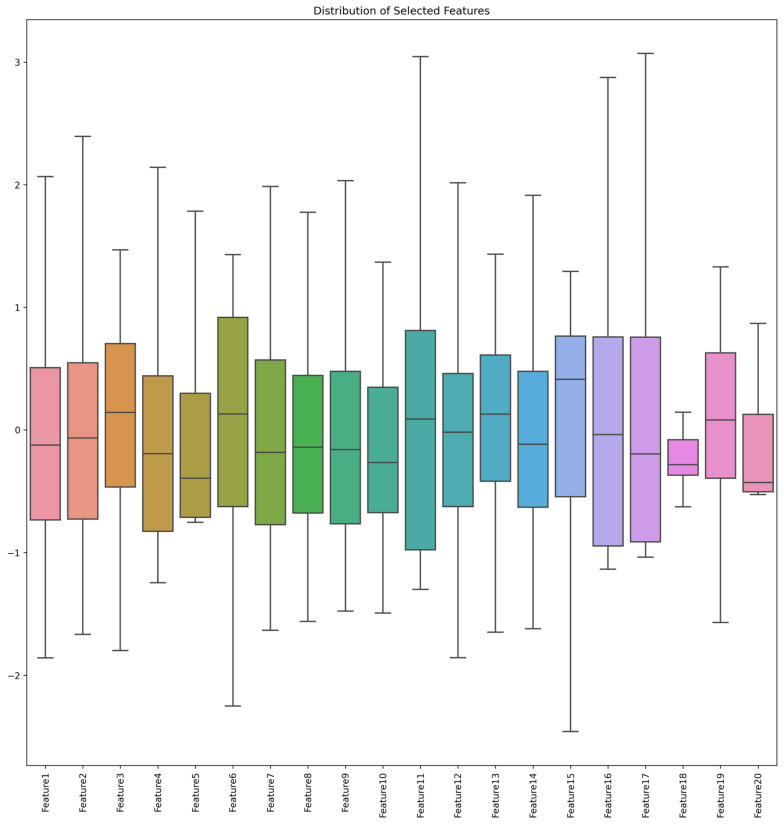
Distribution of selected features.

**Figure 4:**
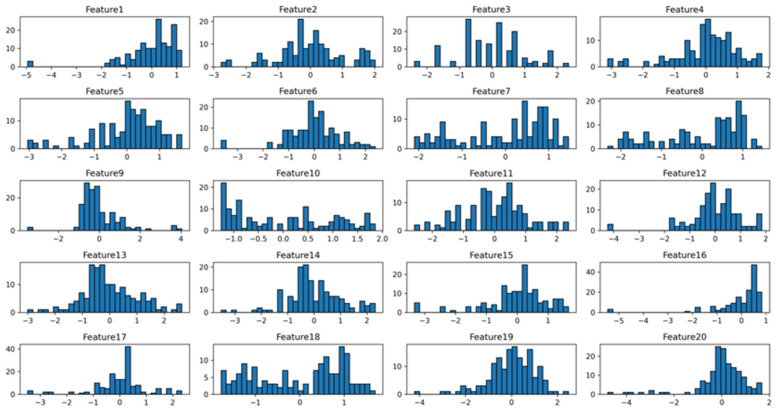
Histogram of dataset distribution.

Using the RFE method, 20 of the most important features were extracted from the original data on knee joint muscle strength and GDI. For ease of subsequent analysis and presentation, these features were renamed to Feature 1 through Feature 20. The mapping between the original feature names and the new names is detailed in Table 4. The distribution of the extracted features is displayed through histograms, as shown in Figure 3 and Figure 4. The data for Features 1 through 20 are evenly distributed and tend to follow a near-normal distribution. This indicates that the dataset’s attributes and labels are stable, with no significant bias or imbalance.

Observing the matrix, we can identify strong positive correlations between certain features. For instance, Features 8 and 9, as well as Features 16 and 17, have correlation coefficients close to 1 (0.97), indicating a very high positive correlation. Similarly, Feature 8 and Feature 14 also exhibit a strong correlation (0.95). These highly positive correlations suggest that these features share similar trends in the dataset and may collectively reflect similar biological or functional information. On the other hand, some features, such as Feature 5 and Feature 6, show a significant negative correlation (−0.37). This indicates that these features have opposite trends in the dataset, potentially representing different or mutually inhibitory physiological mechanisms. Such negative correlations could play a complementary role in model prediction, thereby influencing the overall performance of the model.

**Figure 5:**
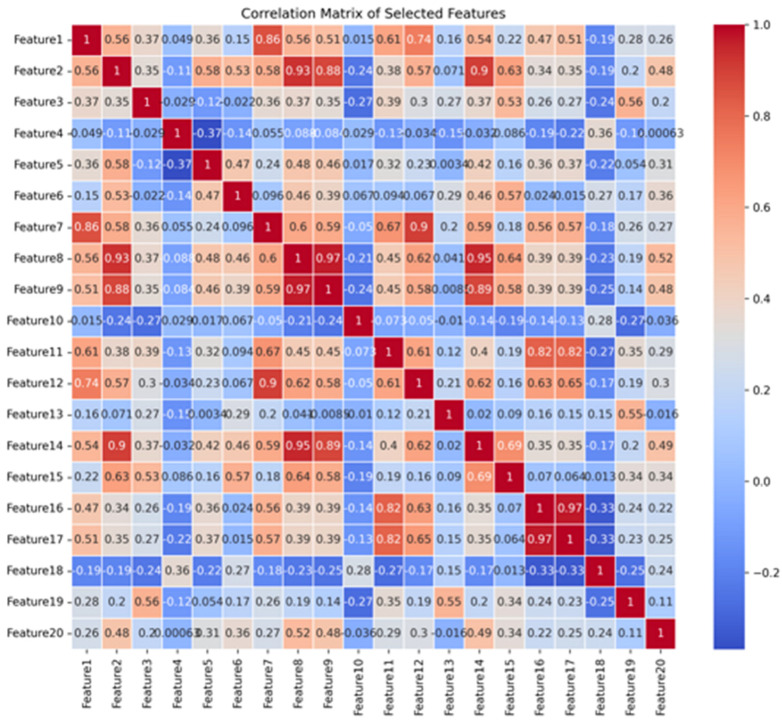
Correlation matrix of selected features. Note: The colors in the matrix range from blue to red, indicating the direction and strength of the correlations between features. Blue represents negative correlations, while red represents positive correlations, with the intensity of the color reflecting the strength of the correlation.

**Figure 6:**
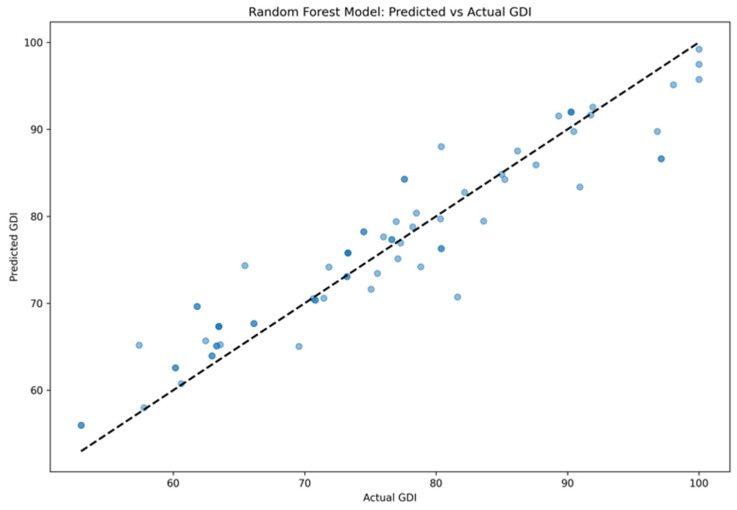
RF model prediction scatterplot. Note: in the plot, the dashed line represents the reference line for perfect prediction (i.e., where the predicted values equal the actual values).

The performance results of the four predictive models are summarized in Table 5. Overall, each model demonstrated varying degrees of effectiveness in predicting the GDI for post-stroke hemiplegic patients, with the Random Forest model showing the best overall performance. Specifically, the Random Forest model had an MSE of 16.18 ± 1.92 and an R^2^ of 0.89 ± 0.06, indicating higher predictive accuracy and reliability. Additionally, it had the lowest MAE of 2.99 ± 0.69. In comparison, the Lasso Regression model, while effective, performed slightly lower with an MSE of 22.29 ± 3.28 and an R^2^ of 0.85 ± 0.18. The SVR model and the BP Neural Network model showed moderate to lower performance, with the SVR model yielding an MSE of 31.58 ± 5.48 and an R^2^ of 0.82 ± 0.13, and the BP Neural Network model exhibiting the poorest performance, with an MSE of 50.38 ± 9.12 and an R^2^ of 0.79 ± 0.21. These results suggest that although all models possess predictive capabilities, the Random Forest model provides the most accurate and consistent results. Figure 5 illustrates a scatter plot comparing the GDI values predicted by the RF model with the actual GDI values. The scatter plot shows that most of the predicted values are close to the dashed line, indicating high predictive accuracy and stability of the model. See Figure 6.

**Figure 7:**
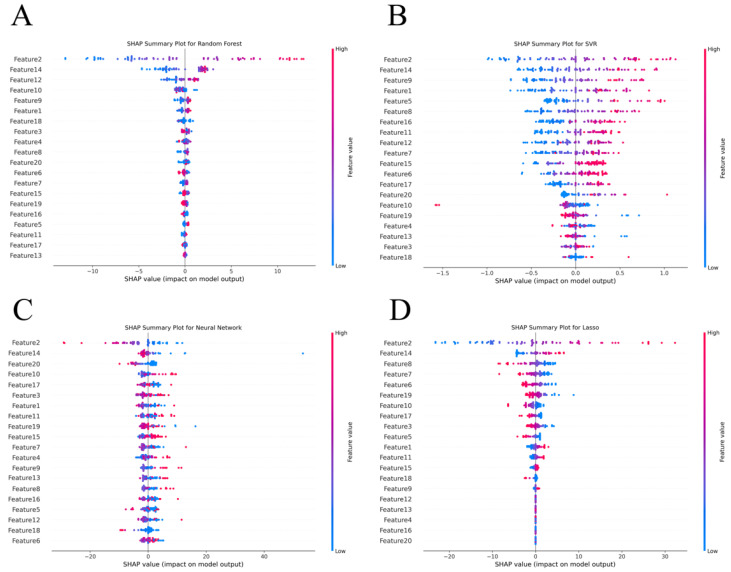
SHAP summary plot of different models. (**A**) RF model results; (**B**) SVR model results; (**C**) BP Neural Network model results; (**D**) Lasso Regression model results. Note: in these plots, the color gradient from blue (low value) to red (high value) represents the size of the feature values, and the *x*-axis represents the SHAP values, with larger values indicating a more significant impact of the feature on the model’s output.

Figure 7A–D present summary plots of SHAP values for the four different models, which help to interpret the decision-making process by showing each feature’s contribution to the model’s predictions. Figure 7A displays the SHAP values for the Random Forest model. It is evident that Feature 2, Feature 14, and Feature 12 have a substantial positive influence on the model’s predictions. Figure 7B illustrates the distribution of feature importance in the SVR model. In this model, Feature 2, Feature 14, and Feature 9 have a strong impact on the model’s output, while features like Feature 18 and Feature 3 contribute less and, in some cases, may even have a negative effect. This indicates that the importance of features in the SVR model is more dispersed. Figure 7C shows the SHAP value distribution for the BP Neural Network model. Here, Feature 2, Feature 14, and Feature 20 significantly influence the model’s output, highlighting their importance in the neural network. Interestingly, compared to other models, Feature 3 and Feature 1 have more prominent SHAP values, suggesting that the BP Neural Network model relies more heavily on certain key features. Figure 7D presents the SHAP value analysis for the Lasso Regression model. In this model, Feature 2, Feature 14, and Feature 4 have high SHAP values, indicating their crucial role in the model’s predictions. Additionally, Feature 8 and Feature 7 also show considerable influence, suggesting that the Lasso Regression model effectively balances the contributions of multiple features.

Notably, across all four models, Feature 2 and Feature 14 consistently exhibit significant influence, underscoring their importance in predicting GDI. This consistency across models highlights the critical role these features play in the prediction process.

The results of this study suggest that in clinical environments with limited resources or smaller datasets, the RF model may be more suitable. This provides clinicians with an efficient and reliable tool for gait prediction, holding significant practical value.

## 4. Discussion

This study aimed to predict the GDI in post-stroke hemiplegic patients by integrating knee joint isokinetic strength assessment data with machine learning models. The results indicate that the RF model performed exceptionally well in handling complex biomechanical data, particularly in predicting gait deviations. This aligns with previous research, which has shown that the RF model can effectively manage high-dimensional data in clinical settings [32]. For example, Correa et al. highlighted the advantages of using RF for predicting clinical outcomes in stroke rehabilitation, demonstrating its reliability and effectiveness in analyzing gait data [33]. Additionally, the RF model’s accuracy and stability significantly outperformed traditional models such as SVR and the BP Neural Network model. Similar findings have been reported by Martello et al., suggesting that ensemble methods tend to generalize better with complex datasets, reducing the risk of overfitting [34]. The RF model, by integrating multiple decision trees, effectively reduced the risk of overfitting, demonstrating greater robustness [29]. RF models have shown advantages in predicting survival rates in chronic obstructive pulmonary disease (COPD) patients and assessing cardiovascular disease risks, showcasing their strength in processing multidimensional clinical data [35]. Moreover, the model’s effectiveness in managing nonlinear relationships and noisy data further supports its applicability and reliability in gait analysis [36].

In contrast, while deep learning models excel in processing imaging data, they tend to overfit with smaller sample sizes, leading to insufficient generalization capabilities [37]. Although deep learning models have demonstrated superior predictive accuracy, their “black-box” nature limits their clinical application [38]. This study identified that Feature 2 (maximum work of the affected side’s knee flexors at 60°/s) and Feature 14 (maximum work of the affected side’s knee extensors at 120°/s) were particularly important for predicting GDI. Previous studies have demonstrated that knee extensor strength is crucial for maintaining gait stability, particularly during the stance phase. For instance, research indicates that individuals with stronger knee extensors exhibit better dynamic postural stability, which is essential for effective ambulation and reducing fall risk [39]. Additionally, findings suggest that deficits in knee extensor strength can lead to impaired stability during weight-bearing activities, which may contribute to gait deviations and an increased likelihood of falls [40]. Moreover, the role of knee extensors in controlling the knee joint during the stance phase is critical, as they help manage the forces acting on the knee and maintain alignment, thereby enhancing overall gait stability [41].

Feature 2 reflects the strength performance of knee flexors at a specific angular velocity, which is crucial during the swing phase of the gait cycle to lift the foot and ensure smooth and stable movement. Feature 14, on the other hand, highlights the critical role of knee extensor strength at higher angular velocities, particularly during the stance phase, in maintaining gait stability and coordination. The existing literature widely supports this finding. Research shows that knee extensors play a crucial role during the stance phase of the gait cycle, particularly when patients need to maintain body stability and bear weight [42]. Insufficient extensor strength is often associated with stance phase issues, potentially leading to hyperextension or flexion deformities of the knee, thereby affecting gait stability and symmetry [43]. Knee flexors, on the other hand, are essential during the swing phase of the gait cycle, where they help in lifting the foot and preparing for the next step. Insufficient flexor strength can result in difficulties with foot clearance, potentially leading to tripping or inefficient gait patterns. Additionally, the differences in extensor and flexor work across different angular velocities reflect muscle performance under dynamic loading conditions, which is especially important for gait control in post-stroke hemiplegic patients [44,45]. Further research suggests that enhancing both knee extensor and flexor strength can effectively improve gait performance, reduce the risk of joint injuries, and enhance walking ability and independence [46]. Meanwhile, insufficient strength in these muscle groups may increase the risk of gait instability, leading to a higher likelihood of falls and recurrent strokes [47]. This study further validates the importance of knee muscle strength in gait control, particularly in the rehabilitation process of post-stroke hemiplegic patients. Strengthening these key muscle groups can significantly improve gait, reduce fall risk, and accelerate recovery, providing a solid scientific basis for developing personalized rehabilitation plans [42,48].

The use of SHAP technology significantly enhanced the interpretability of complex models by providing consistent and globally interpretable feature contribution values, which is also of great significance in clinical applications. For example, SHAP technology has been successfully applied in cardiovascular risk prediction models, increasing doctors’ confidence in model results [49]. Similarly, SHAP’s application in diabetes management has not only improved model interpretability but also helped in designing more precise personalized treatment plans [50]. By introducing SHAP technology, this study further improved the clinical applicability of machine learning models, making prediction results easier for doctors to understand and apply, thereby optimizing the development of personalized rehabilitation plans [51].

In handling small sample sizes, traditional machine learning models demonstrated significant advantages [52]. BP Neural Networks are prone to overfitting with small samples, resulting in inadequate generalization capabilities. For example, the literature points out that deep learning models often exhibit instability when processing small-sample data, requiring regularization or data augmentation techniques for improvement [53]. In contrast, ensemble learning models such as Random Forest are more suitable for handling complex biomechanical data with small sample sizes, as they better manage data noise and avoid overfitting [54]. Moreover, studies have shown that traditional machine learning models often provide more robust predictive results when handling high-dimensional data, especially when the sample size is limited, further supporting the findings of this study [55].

Despite the significant achievements in predicting gait deviations, this study has some limitations. First, the sample size was relatively small, including only 150 post-stroke hemiplegic patients. Although this sample size provided initial validation for model development, its generalizability might be limited when dealing with more complex and diverse patient populations. Future research should expand the sample size and include a more diverse patient population, encompassing different genders, ages, disease courses, and rehabilitation stages. By increasing sample diversity, researchers can develop more generalizable gait prediction models, improving model generalization ability and providing more personalized rehabilitation recommendations for different types of patients. Second, this study primarily relied on knee joint isokinetic strength assessment data. While these data are important for gait prediction, gait deviations result from the interaction of multiple factors, including muscle strength, balance ability, neural control, and external environmental factors. Solely relying on knee joint strength data may not fully capture all the relevant factors of gait deviation. Future research should explore the integration of multimodal data, combining balance ability tests, surface electromyography, gait imaging data, neuroimaging data, and biomarker data, to construct a more comprehensive gait prediction model. Integrating multimodal data will help capture the causes of gait deviations more comprehensively, improving the accuracy of the model’s predictions and providing more precise rehabilitation guidance. Finally, although this study used SHAP technology to enhance model interpretability, machine learning models may still exhibit a “black-box” effect in practical applications. This issue is particularly important in clinical decision making, as doctors need to understand the specific basis for model predictions to better apply them in patient rehabilitation plans. Future research should continue to explore how to improve the interpretability of machine learning models, developing more intuitive visualization tools and explanatory methods, making it easier for clinicians to understand and apply these models’ predictions. This is crucial for better integrating machine learning models into the clinical decision-making process.

## 5. Conclusions

In conclusion, this study successfully predicted the GDI in post-stroke hemiplegic patients by integrating knee joint isokinetic strength assessment data with machine learning models. These findings confirm that the RF model is particularly effective in predicting gait deviations, demonstrating significant accuracy and robustness. Future research should focus on addressing potential biases and expanding the sample size to validate these findings further.

## Figures and Tables

**Table 1:** Hyperparameters of each algorithm model.

Method	Parameter	Setting Value
RFE	Model Type	Linear regression (L1 regularization)
	Number of Selected Features	20
	Number of Iterations	10
	Step Size	2
Lasso Regression	α (Regularization Strength)	0.01
	Max Iterations	2000
	Random State	21
BP Neural Network model	Hidden Layers	4 layers, 12 neurons each
	Activation Function	ReLU
	Optimizer	Adam
	Learning Rate	0.0005
	Batch Size	64
	Training Epochs	300
RF	Number of Trees	150
	Max Depth	20
	Min Samples Split	5
	Random State	21
SVR	Kernel Function	RBF
	Regularization Parameter C	0.5
	ε	0.05
	Max Iterations	2000

**Table 2:** Demographic of Stroke Patients.

Characteristic	Study Group (*n* = 150)	Literature Group	Chi-Square Test Result (*p*-Value)
Age Distribution			0.81
40–49 years	37 cases (24.7%)	20.70%	
50–59 years	29 cases (19.3%)	19.70%	
60–69 years	26 cases (17.3%)	17.50%	
70–79 years	8 cases (5.3%)	9.30%	
80 years and above	3 cases (2.0%)	2.80%	
Gender Ratio			0.16
Male	85 cases (56.7%)	45.70%	
Female	65 cases (43.3%)	54.20%	
Residence Distribution			0.75
Urban	88 cases (58.7%)	55.40%	
Rural	62 cases (41.3%)	44.50%	
Hypertension Rate	55 cases (36.7%)	35.24%	0.78
Diabetes Rate	18 cases (12.0%)	9.55%	0.38

**Table 3:** Isokinetic muscle strength test data situation.

Speed (Deg/s)	Muscle Group	Measurement Side	PT (Nm)	PT/BW (%)	Max Work of Repeated Actions (J)	CV (%)	Average Power (W)	Total Work (J)	Acceleration Time (s)	Deceleration Time (s)	ROM (Deg)	Average Peak Torque (Nm)
60	Extensor	Healthy Side	29.03 (43.15, 72.18)	38.45 (64.12, 102.57)	30.42 (51.38, 81.8)	22.0 (10.9, 32.9)	17.45 (21.85, 39.3)	155.15 (190.62, 345.78)	60.0 (60.0, 120.0)	60.0 (120.0, 180.0)	13.55 (99.03, 112.58)	25.72 (33.12, 58.85)
60	Extensor	Affected Side	21.27 (25.43, 46.7)	32.75 (34.56, 67.31)	27.6 (23.6, 51.2)	42.3 (14.3, 56.6)	11.92 (12.28, 24.2)	106.0 (80.9, 186.9)	100.0 (70.0, 170.0)	60.0 (130.0, 190.0)	25.08 (79.38, 104.45)	14.6 (20.36, 34.95)
60	Flexor	Healthy Side	17.48 (15.07, 32.55)	24.94 (22.32, 47.26)	26.9 (8.1, 35.0)	45.9 (9.95, 55.85)	9.88 (2.9, 12.78)	99.8 (24.0, 123.8)	100.0 (90.0, 190.0)	170.0 (120.0, 290.0)	17.3 (94.0, 111.3)	16.5 (11.11, 27.6)
60	Flexor	Affected Side	8.98 (8.81, 17.79)	15.39 (12.79, 28.18)	13.77 (0.4, 14.17)	49.43 (22.47, 71.9)	4.91 (0.1, 5.01)	40.9 (1.6, 42.5)	80.0 (110.0, 190.0)	110.0 (140.0, 250.0)	25.6 (83.1, 108.7)	7.37 (5.77, 13.14)
90	Extensor	Healthy Side	25.12 (37.92, 63.05)	33.42 (58.2, 91.62)	29.83 (46.3, 76.12)	15.02 (7.48, 22.5)	21.27 (30.68, 51.95)	111.2 (206.4, 317.6)	40.0 (80.0, 120.0)	40.0 (120.0, 160.0)	11.25 (101.9, 113.15)	24.73 (31.95, 56.68)
90	Extensor	Affected Side	12.84 (12.41, 25.24)	17.16 (19.94, 37.1)	23.45 (4.45, 27.9)	26.65 (12.35, 39.0)	12.35 (2.3, 14.65)	94.5 (11.82, 106.33)	107.5 (112.5, 220.0)	90.0 (130.0, 220.0)	12.12 (100.5, 112.62)	9.54 (10.28, 19.81)
90	Flexor	Healthy Side	5.97 (7.9, 13.88)	8.05 (11.73, 19.78)	9.18 (0.33, 9.5)	35.98 (18.42, 54.4)	6.06 (0.1, 6.16)	28.77 (0.93, 29.7)	50.0 (120.0, 170.0)	70.0 (130.0, 200.0)	28.1 (81.6, 109.7)	5.33 (6.37, 11.7)
90	Flexor	Affected Side	24.58 (31.9, 56.48)	34.37 (49.52, 83.89)	21.5 (47.4, 68.9)	12.4 (7.1, 19.5)	24.05 (35.12, 59.18)	99.8 (196.8, 296.6)	50.0 (90.0, 140.0)	40.0 (130.0, 170.0)	11.27 (102.2, 113.47)	17.74 (31.21, 48.95)
120	Extensor	Healthy Side	12.92 (25.27, 38.2)	18.35 (37.92, 56.27)	23.95 (23.95, 47.9)	15.68 (6.83, 22.5)	21.79 (19.46, 41.26)	115.03 (92.38, 207.4)	70.0 (130.0, 200.0)	60.0 (150.0, 210.0)	26.05 (83.85, 109.9)	9.84 (22.72, 32.55)
120	Extensor	Affected Side	10.4 (11.97, 22.38)	14.12 (17.32, 31.45)	19.8 (2.3, 22.1)	24.18 (15.93, 40.1)	14.25 (1.55, 15.8)	80.55 (6.45, 87.0)	67.5 (140.0, 207.5)	77.5 (140.0, 217.5)	11.2 (101.6, 112.8)	9.14 (9.05, 18.19)
120	Flexor	Healthy Side	4.59 (9.42, 14.01)	7.04 (13.08, 20.12)	6.83 (0.3, 7.12)	32.3 (24.6, 56.9)	4.7 (0.1, 4.8)	26.83 (1.0, 27.83)	67.5 (142.5, 210.0)	80.0 (140.0, 220.0)	24.5 (85.4, 109.9)	5.2 (6.3, 11.5)
120	Flexor	Affected Side	29.03 (43.15, 72.18)	38.45 (64.12, 102.57)	30.42 (51.38, 81.8)	22.0 (10.9, 32.9)	17.45 (21.85, 39.3)	155.15 (190.62, 345.78)	60.0 (60.0, 120.0)	60.0 (120.0, 180.0)	13.55 (99.03, 112.58)	25.72 (33.12, 58.85)

**Table 4:** Feature mapping table.

New Feature Name	Original Feature Name
Feature1	60deg_ext_healthy_max_work
Feature2	60deg_ext_affected_max_work
Feature3	60deg_flex_healthy_rom
Feature4	60deg_flex_affected_cv
Feature5	60deg_flex_affected_total_work
Feature6	60deg_flex_affected_rom
Feature7	90deg_ext_healthy_max_work
Feature8	90deg_ext_affected_max_work
Feature9	90deg_ext_affected_total_work
Feature10	90deg_ext_affected_dec_time
Feature11	90deg_flex_healthy_max_work
Feature12	120deg_ext_healthy_total_work
Feature13	120deg_ext_healthy_rom
Feature14	120deg_ext_affected_max_work
Feature15	120deg_ext_affected_rom
Feature16	120deg_flex_healthy_max_work
Feature17	120deg_flex_healthy_total_work
Feature18	120deg_flex_healthy_acc_time
Feature19	120deg_flex_healthy_rom
Feature20	120deg_flex_affected_total_work

**Table 5:** Performance results of different models.

Model	MSE	R^2^	MAE
Lasso Regression	22.29 ± 3.28	0.85 ± 0.18	3.71 ± 0.96
Random Forest	16.18 ± 1.92	0.89 ± 0.06	2.99 ± 0.69
SVR	31.58 ± 5.48	0.82 ± 0.13	7.68 ± 1.70
BP Neural Network model	50.38 ± 9.12	0.79 ± 0.21	9.59 ± 1.99

## Data Availability

Data are contained within the article.
